# Harnessing eCISs for precision phytomicrobiome engineering and biocontrol

**DOI:** 10.1093/femsre/fuag006

**Published:** 2026-02-27

**Authors:** Gunarathna R D S Madushani, Xue Wu, Wikum H Jayasinghe, Qi Wang, Kumar Vinit, Ge-Fei Hao

**Affiliations:** State Key Laboratory of Green Pesticide, Center for Research and Development of Fine Chemicals of Guizhou University, Guiyang 550025, P.R. China; State Key Laboratory of Green Pesticide, Center for Research and Development of Fine Chemicals of Guizhou University, Guiyang 550025, P.R. China; Department of Agricultural Biology, Faculty of Agriculture, University of Peradeniya, Peradeniya 20400, Sri Lanka; State Key Laboratory of Public Big Data, College of Computer Science and Technology, Guizhou University, Guiyang 550025, P.R. China; State Key Laboratory of Green Pesticide, Center for Research and Development of Fine Chemicals of Guizhou University, Guiyang 550025, P.R. China; State Key Laboratory of Green Pesticide, Center for Research and Development of Fine Chemicals of Guizhou University, Guiyang 550025, P.R. China; State Key Laboratory of Green Pesticide, Central China Normal University, Wuhan 430079, P.R. China

**Keywords:** extracellular contractile injection systems, tailocins, phytomicrobiome engineering, biocontrol, targeted specificity, payload delivery

## Abstract

Plant microbiome disruption often increases vulnerability to crop diseases, endangering worldwide food production, while chemical pesticides become increasingly less viable and continue to damage ecosystems. To safeguard plant microbiome health, several biological control strategies offer alternatives, yet many operate through broader or weakly defined target mechanisms. In recent years, bacterial contractile injection systems (BCISs) have emerged as a promising class of naturally evolved nanomachines that translocate molecular payloads directly into target cells. Subsets of these systems, extracellular contractile injection systems (eCISs), are distinguished by their specific narrow host range and receptor-dependent specificity. Recent studies have demonstrated that eCISs provide a transformative approach for targeted microbial manipulation, enabling the delivery of specialized molecules into particular microbes with higher precision. However, despite their potential, the integration of these engineered injection systems with microbial modulation for phytomicrobiome remains largely underexplored. Here, we explore the capabilities of eCISs as an advanced approach for the biocontrol, leveraging their tailored mechanisms for targeted payload delivery in plant-associated microbial communities with enhanced host specificity. This study aims to address the potential of engineered injection systems in facilitating sustainable phytomicrobiome engineering strategies that enhance biocontrol, aiming to reduce environmental harm while improving agricultural productivity.

## Teaser

Engineered extracellular contractile injection systems (eCISs) provide an ecofriendly, precision alternative to chemical pesticides, selectively targeting harmful crop pathogens while safeguarding beneficial microbes to improve plant health and sustainable agriculture.

## Introduction

The plant microbiome represents a rich repository of microbiome-related genomic potential, which helps to sustain host health and diverse ecosystem functions (Henry 2025). Such a complex network of bacteria, fungi, archaea, protists, and viruses inhabits from the rhizosphere to the endosphere, controls crucial processes, including nutrient cycling and degradation of pollutants, which in turn promote sustainable plant growth and resilience (Levy et al. 2018, Compant et al. 2019). Anthropogenic disturbance through intensive agriculture, monoculture culture of crops, and heavy use of chemical pesticides has reduced stability in these microbial communities (French et al. 2021). For example, pests and pathogens cause above US$40 billion annual global crop losses, a toll aggravated by a lack of efficacy in conventional chemical treatments (Arif et al. 2020). Such disturbances hence reduce functional diversity, compromise the fertility of the soil, and weaken plant immunity against the invasion of pathogenic organisms and abiotic stress (French et al. 2021, Peng et al. 2024). Therefore, plant microbiome engineering has emerged as a nature-based strategy by taking deliberate and precise manipulations of microbial communities associated with plants (Arif et al. 2020). Thus, an ability to tap into the potentials of phytomicrobiome engineering offers a promising avenue for restoring the ecological balance and safeguarding resilient agricultural systems.

The plant microbiome engineering leverages the notion of precise manipulation of functional capabilities of microbes that control nutrient cycling, organic matter degradation, and plant immune system functions (Patyal et al. 2025). The studies have shown advanced techniques including omics analyses, gene editing, and community sequencing have revealed microbial species and their genes influencing plant performance (Wagner et al. 2016, Trivedi et al. 2020, Ke et al. 2021). Indeed, such findings enable the rational design of synthetic microbial consortia (SynComs) displaying persistent growth-promoting and stress-mitigating functions (Castrillo et al. 2017, Sharma and Bora 2025). Also, engineered microbial strains further monitor plant stress signals, confer growth-promoting functions, and silence pathogen genes via RNA interference, which enables the accurate and controlled suppression of crop diseases (Ganbaatar et al. 2017). By contrast, the efficiency of conventional agricultural chemicals is still limited since more than 90% of the applied agricultural chemicals fail to target the desired pest effectively (Arif et al. 2020). Moreover, the widespread development of antibiotic resistance further limits the potential for broad-spectrum control approaches (Ruuskanen et al. 2023). In this case, the application of narrow-spectrum antimicrobial represents the exact strategy to inhibit the microbes and ensure the safety of beneficial microbes.

Highly targeted microbial antagonism is an emerging powerful strategy for precision-based plant protection, where bacterial contractile injection systems (BCISs) represent a promising tool in this context. Emerging studies have indicated that BCISs, phage tail-like particles with specific receptor recognition, can be harnessed for highly targeted pathogen suppression. These are bacterial cell–cell interactions through sophisticated macromolecular machines, which translocate effectors either into the extracellular medium or directly into target cells (Lin 2024). Based on their distinct modes of action, BCISs are classified into intracellular type VI secretion systems (T6SS) and extracellular contractile injection systems (eCISs), which are highly versatile and widespread among both Gram-negative and Gram-positive bacteria (Xu et al. 2022). Recent breakthroughs identify tailocins as a major eCIS family, comprising rigid, contractile R-type nanotubes resembling myovirus tails and flexible, noncontractile F-type rods (Backman et al. 2024a, Marín-arraiza et al. 2025). Other significantly characterized eCISs include antifeeding prophages (Afps), Photorhabdus virulence cassettes (PVCs), and metamorphosis-associated contractile structures (MACs) (Jiang et al. 2019). To date, above 1400 eCIS loci have been identified across various bacterial and archaeal genomes, enabling programmable effector delivery, along with the antibacterial and eukaryotic toxicity (Geller et al. 2021). Despite this potential, a comprehensive review linking eCIS mechanisms to their emerging roles in plant-associated microbial communities is still lacking.

Here, we discuss how eCISs function as a promising phytomicrobiome engineering tool, exploiting their programmable delivery capabilities for targeted manipulation and biocontrol of phytopathogens (Backman et al. 2024b). We also explore how eCISs could revolutionize plant microbiome manipulation through three key avenues: (1) advancing precision manipulation of plant microbiomes, (2) reprogrammable eCIS as tool for precision plant microbial modulation, and (3) eCIS-based precision plant microbial modulation as innovative biocontrol approach (Rajaure et al. 2015, Kreitz et al. 2023, Zachs et al. 2024). Therefore, this review emphasizes the foundational insights into eCIS-based precision microbiome manipulation, enabling targeted phytopathogen biocontrol while preserving ecosystem integrity.

## Advancing precision manipulation of plant microbiomes

The precision manipulation of plant-associated microbiome represents specific modulation of the microbial composition and diverse functions without causing disturbances to the beneficial microbes. This contrast to broad-spectrum interventions, indiscriminately affect entire microbial communities, very often at the expense of ecosystem resilience and beneficial function. The central goal is focused on selectively promoting or inhibiting certain microbes or metabolic processes while maintaining the network of ecosystems for plant health and productivity (Zhou et al. 2024). Such precision demands thorough understanding of functional dynamics of microbes associated with plants and development of engineering techniques that will enable predictable and controlled manipulations (Compant et al. 2019). Microbial inoculants, synthetic communities, host genotype-informed recruitment, and narrow-spectrum antimicrobial strategies have emerged as complementary approaches, offering different levels of precision in driving beneficial functions from nutrient mobilization through pathogen suppression. Recent studies have shown that certain microorganisms or processes stimulate or suppress specific microorganisms with no effects on overall microbial stability (Iralu et al. 2024, Czajkowski and Matilla 2025 et al. 2025). These emerging technologies, collectively, make possible the rational engineering of plant microbiomes with unprecedented control, facilitating predictable, resilient, and ecologically informed outcomes in agricultural systems (Orozco-mosqueda et al. 2018). This section examines the progression from broad foundational approaches to increasingly precise manipulation strategies in plant microbiome engineering.

### Functional dynamics of plant-associated microbial communities

**Figure 1:**
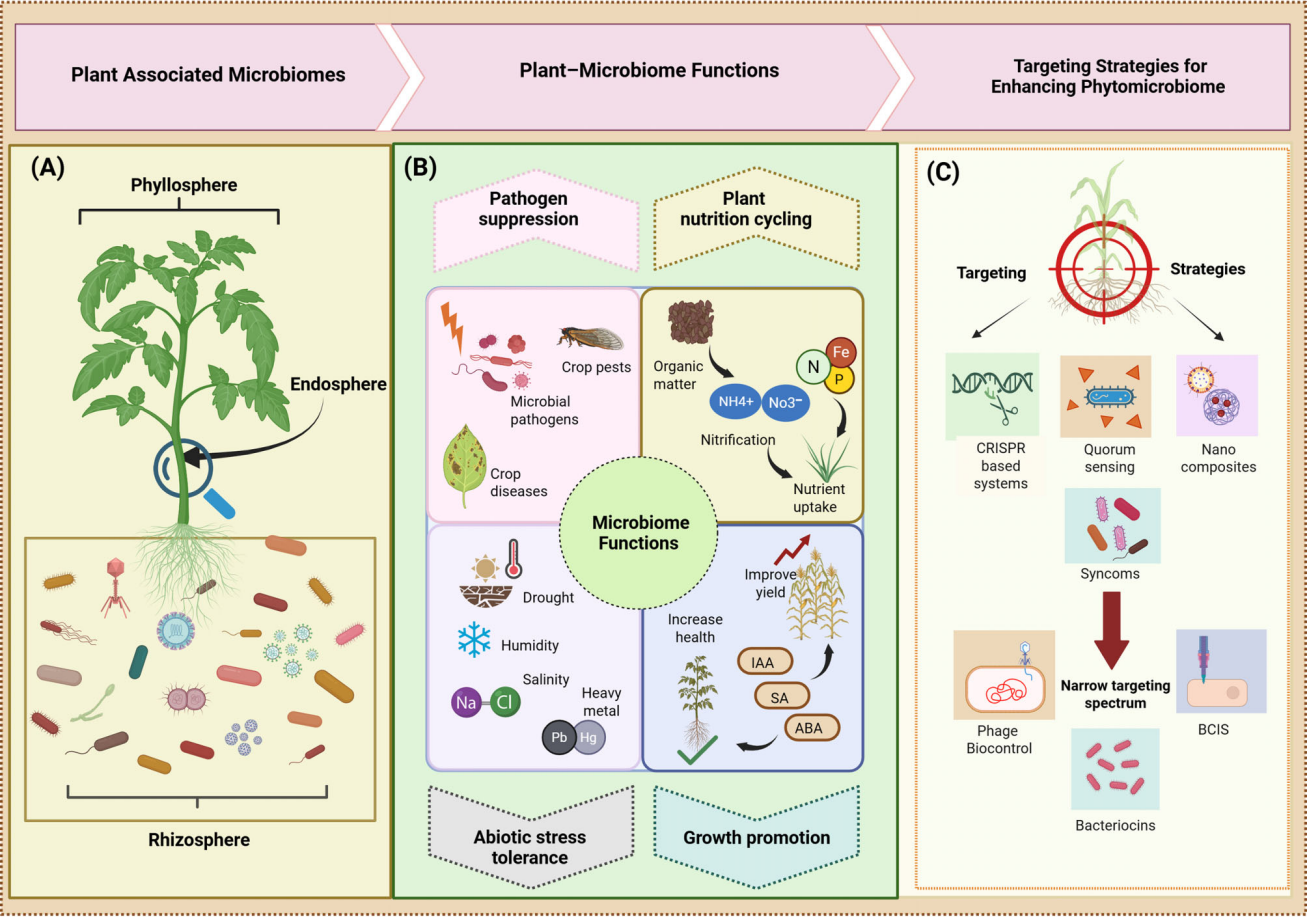
The hierarchical organization of core plant microbial community functions and precision microbial targeting strategies. (A) Overview of core plant microbial community components, such as the phyllosphere, endosphere, and rhizosphere. (B) Primary applications of microbiome manipulation include pathogen suppression, nutrient cycling, enhancing stress resilience, and promoting plant growth. (C) Evolution of microbial targeting strategies for enhancing phytomicrobiomes, ranging from CRISPR systems, SynComs, quorum sensing, nanocomposites to targeted approaches such as phage therapy, bacteriocins, and BCIS, which enable precise modulation of plant-associated microbial communities.

Plants coexist with diverse populations of microorganisms, which help to assist in growth, health, and stress resilience. Microbial community composition depends on both biotic factors plant (species and developmental stage) and abiotic factors (climate and soil physico-chemistry) (Marian et al. 2022). These microorganisms reside in different plant compartments: the rhizosphere, phyllosphere, and endosphere, and are composed of bacteria, fungi, viruses, protists, and nematodes (Trivedi et al. 2020) (Fig. 1A). The functional diversity of plant-associated microbiota runs from neutral to pathogenic or beneficial and impacts nutrient acquisition, plant growth, pathogen defense, and abiotic stress resistance (Saad et al. 2020). Quantitatively, bacterial and fungal taxa dominate, although archaea and protists also play critical roles in soil biogeo-chemical cycling and ecosystem functioning (Ren et al. 2019, Farrar et al. 2014). Microbial composition and function vary substantially across plant tissues and environmental niches, reflecting the spatial and functional complexity of plant microbiomes. Understanding these dynamics provides the foundation for the development of targeted approaches that enable the controlled manipulation of microbiome composition and function.

Recent advancements in sequencing techniques have unveiled a large variation in microbiome composition among plant tissues (Wang et al. 2022). For example, rhizosphere communities are majorly composed of Proteobacteria, while the endosphere is enriched with both Firmicutes and Proteobacteria. Phyllosphere microbiome is dominated by Bacteroidetes, Actinobacteria, Proteobacteria, and Firmicutes. Fungal populations in plant microbiome are mainly consists of Ascomycota and Basidiomycota (Ahmed et al. 2023, Ling et al. 2022). Within these microbiomes, bacteria live in symbiotic, parasitic, and mutualistic relationships, where parasitic bacteria lead to yield loss. Mutualistic nitrogen-fixing bacteria found in legumes are the basis for biofertilizer approaches that improve plant growth (Tsiknia et al. 2020). Antagonistic microbes within the phytobiome provide biological control potential by producing bioactive compounds that suppress pathogens and reduce crop damage.

The plant microbiome supports host defense through direct antimicrobial activity and immune modulation. Many microbial taxa produce secondary metabolites, antibiotics, lipopeptides, chitinases, that inhibit pathogen growth or cause cellular damage (Zhang et al. 2023, Demisie et al. 2024). *Bacillus thuringiensis* (Bt) produces Cry proteins that selectively disrupt lepidopteran midgut cells; Bt-based formulations have largely replaced organophosphorus insecticides, significantly reducing chemical residues while controlling corn borers and bollworms (Lázaro-berenguer et al. 2024). Beyond direct antagonism, root-associated microbes enhance host immunity by activating induced systemic resistance (ISR) (Pieterse et al. 2009). Genera such as *Rhizobia* and *Bacillus* spp. triggers salicylic and Jasmonic acids signaling pathways, providing broad-spectrum systemic resistance against pathogens (Vieira et al. 2024, Hashem et al. 2019). Furthermore, SynComs comprising selected antagonistic and plant growth-promoting microbes represent an emerging biological control strategy enabling cooperative disease suppression (Northen et al. 2024). A SynCom comprising *Pseudomonas* sp. NA13 and *Trichoderma* spp. achieved 90% control of tomato Fusarium wilt, outperforming single-strain agents (Zhou et al. 2022). This microbial-driven functionality builds a biological protection system offering potential to reduce current overreliance on chemical pesticides.

The plant microbiome also plays essential roles in nutrient acquisition and stress resistance (Fig. 1B). Nitrogen-fixing bacteria such as *Azospirillum* spp. enhance nitrogen use efficiency in maize and wheat crops, increasing yields up to 10%–15% (Cassán and Diaz-Zorita 2016). Furthermore, phosphate-solubilizing bacteria including *Burkholderia* species increase soil phosphorus availability by 50%–80%, while *Paenibacillus mucilaginosus* mobilizes potassium, enhancing apple potassium uptake by 30% in deficient soils (Ma et al. 2025, Chen et al. 2020). Siderophores produced by *Pseudomonas fluorescens* triple iron absorption in alkaline soils, alleviating iron deficiency chlorosis (Schalk 2025). The microbiome increases tolerance to abiotic stresses, drought, salinity, and heavy metal toxicity, by modulating their molecular pathways (Ahmed et al. 2023). In *Cicer arietinum*, treatment with proline-producing *Bacillus subtilis* and *B. thuringiensis* increased leaf relative water content (Khan et al. 2019). *Bacillus amyloliquefaciens* promotes lateral root growth in wheat, improving salt stress survival by 40% (Ji et al. 2022, Li et al. 2025a). Also, root-associated microbiota releases organic acids that chelate toxic Cd²⁺ and Pb²⁺ ions, enhancing plant tolerance (Podar and Maathuis 2021). These specific interactions emphasize the phytomicrobiome as a dynamic biological system facilitating plant productivity and healthy ecosystem performance.

### Established approaches toward precision–phytomicrobiome engineering

Plant-associated microbiome offers economical solutions to sustainability challenges in agricultural systems, though earlier approaches varied in their precision and diverse ecological impact. Initial strategies focused on primarily single-strain inoculants, rhizobia, mycorrhizae, nitrogen fixers, entomopathogens, endophytes, and plant growth-promoting rhizobacteria (Das et al. 2025). By utilizing well-adopted symbioses, including grain legume–rhizobia and root–mycorrhizae associations, these inoculants were normally applied for the seed treatments, soil drenches, or foliar sprays to provide specific plant benefits (Nakei et al. 2022, Das et al. 2025). However, single-strain inoculants often provided inconsistent field results despite promise in limited controlled environments. To overcome these limitations and achieve more controlled plant protection, emerging techniques have been developed that account for intermicrobial dynamics and host–microbe interactions. Therefore, strategies including host-directed engineering, SynComs, nanocarrier delivery, quorum-sensing modulation, and bacteriophage engineering approaches enable increasingly targeted manipulation, though they vary considerably in their precision and selectivity (Bettenworth et al. 2022, Ruan et al. 2024, Lowry et al. 2024, Compant et al. 2025; Fig. 1C). Collectively, these approaches represent a progression toward the precision manipulation rather than fully precise tools themselves, offering varying degrees of control over microbial community structure and function.

Breakthroughs in microbial ecology have shifted focus toward the understanding interspecies dynamics within various microbial communities. For example, cooperative microbes like *Delftia* and *Azospirillum* species, depend on the host plant genetics for their diverse functional expression (Remans et al. 2008). This insight illustrates the importance of host–microbe and microbe–microbe interactions in determining the efficacy of microbial inoculants. Advances in microbiome research have revealed beneficial microbial consortia in disease-suppressive soils, where specific synergistic interactions between dominant taxa contribute to crop health performance. Metagenomic analysis demonstrates that pathogen-induced enrichment of *Chitinophagaceae* and *Flavobacteriaceae* in the root endosphere activates chitinase and NRPS-PKS gene clusters, providing disease suppression through synergistic microbial consortia (Carrión et al. 2019). Identifying essential features of these complex microbial ecosystems remain still challenging. One promising strategy involves designing SynComs, which improve development efficiency of beneficial microbial consortia.

High-throughput screening techniques and SynCom-based development have enabled reconstruction of simplified yet functionally enriched microbiomes. These approaches have demonstrated key microbial interactions and facilitated the design of microbial consortium-derived biofertilizers, biopesticides, and biostimulants for sustainable agricultural applications (Jing et al. 2024, Pandey and Singh 2025). A well-defined microbial consortium including *Comamonas testosteroni, Pseudomonas putida, Enterobacter cloacae*, and *Citrobacter freundii*, doubled crop productivity by enhancing phosphate mobilization (Baas et al. 2016). Furthermore, cross-kingdom symbiosis between *Rhizophagus irregularis* and *Azotobacter vinelandii* significantly improved root expansion, demonstrating the advantages of multispecies microbial networks (Arif et al. 2020). Therefore, these findings demonstrate a transformative shift from single-organism applications toward the design and deployment of microbial networks.

Emerging studies on rhizosphere signaling has emphasized molecular and various biochemical interactions between plants and symbiotic microorganisms. These signaling molecules influence diverse organisms, including plants themselves (Wang et al. 2022). Interkingdom communication between plants and mycorrhizal or rhizobial microbiota is crucial for the symbiosis establishment, while recent evidence of signaling with nonsymbiotic microorganisms highlights broader roles of communication in shaping the phytomicrobiome. Many microorganisms synthesize various quorum-sensing molecules such as *N*-acyl homoserine lactones, diffusible signal factors, to regulate coordinated community behaviors (Hartmann et al. 2014). Beyond microbial communication, these quorum-sensing molecules provide as interkingdom signaling agents that recognized by plants, regulating plant development and activating immune responses including ISR and systemic acquired resistance (Compant et al. 2025). Development of orthogonal quorum-sensing systems represents a major innovation, allowing engineered strains to exchange signals without cross-talk with their native populations. These insights underscore potential for manipulating microbial signaling pathways to modulate the rhizosphere microbiome for enhanced plant growth, immunity, and stress resilience, though such approaches still affect relatively broad microbial populations.

As a promising strategy, nanomaterials provide complementary tools for targeted manipulation with varying degrees of precision. Tailored nanomaterials increase crop productivity while reducing various chemical inputs through precision delivery systems and sustained release, offering an ecofriendly alternative to conventional agrochemicals (Sodhi et al. 2025). For example, Ahmed et al. (2023) developed chitosan–iron nanocomposites that regulate the rhizosphere microbiome structure, reducing the incidence of rice bacterial blight by 50%. Star-shaped polymeric nano-carriers (37.3% loading) deliver naringenin to suppress rhizosphere pathogens, improving soil mobility, antimicrobial efficacy (41% control), and plant resistance while minimizing environmental impact (Su et al. 2023). Despite their diverse agricultural benefits, these nanomaterials increase concerns based on their potential of molecular-level disruptions (enzymatic, photosynthetic, and root dysfunction) and ecosystem risks through soil or water accumulation process and food chain transfer conditions (Murali et al. 2022). These insights underscore potential for manipulating microbial signaling pathways to modulate the rhizosphere microbiome for enhanced plant growth, immunity, and stress resilience, though such approaches still affect relatively broad microbial populations.

### Narrow-spectrum strategies for precision manipulation of phytomicrobiomes

While 20th-century broad-spectrum antibiotics revolutionized disease management in clinical and veterinary contexts and concerns over resistance gene spread have limited their agricultural application, especially against the phytopathogens (Dyary 2023 et al. 2023, Batuman et al. 2024). This has driven the development of narrow-spectrum strategies that can precisely target a specific microbial population, while maintaining the beneficial microbial functions of the phytobiome (Batuman et al. 2024). Therefore, bacteriophages, antimicrobial peptides (AMPs), and BCISs represent distinct approaches to achieving this higher precision, each with unique mechanisms and specificity profiles (Fig. 1C).

Bacteriophages impose a strong selective pressure on microbial communities and have a highly specific bactericidal activity that can be utilized for targeted killing (Koskella and Taylor 2018). While numerous model phages are characterized by narrow host ranges, emerging ecological and metagenomic evidence indicates that phage specificities may span from narrow to broad. Host-range modulation may be influenced by adaptations in the host receptor-binding proteins (RBPs), as well as by proteins involved in other stages of the phage life cycle (Jonge et al. 2019, Smith et al. 2023). Phage biocontrol have proved effective in combating *Ralstonia solanacearum*, substantially reducing wilt incidence in tomato cropping systems (Magar et al. 2022). Moreover, bacteriophage ACPWH, with broad-spectrum properties against *Acidovorax citrulli*, shows great promise as an inexpensive biocontrol agent for bacterial fruit blotch in watermelon (Rahimi et al. 2020). Bacteriophages FoX2 and FoX6 efficiently infect *Xanthomonas campestris* pv. by binding specific cell wall polysaccharides, thus providing sustainable and field-proven biocontrol against the black rot in *Brassica* crops (Fortuna et al. 2023). Despite advances in pathogen suppression, phage biocontrol faces challenges because lytic phages can unpredictably alter microbial community structure, affect bacterial abundance and diversity, and disrupt host-associated microbiomes during ecological interactions (Morella et al. 2018). Phages can replicate and restructure soil microbial communities, potentially driving resistance or destabilizing microbiomes. Also, rigorous biosafety evaluation is essential before their registration and commercial deployment as plant-protection products (Holtappels et al. 2021). Specifically, these phage-related components offer targeted alternatives for plant disease control, further research is necessary to assess their effectiveness at a microbiome scale.

As alternatives to phage biocontrol, AMPs provide narrow-spectrum targeting with distinct advantages. AMPs represent diverse classes of proteinaceous compounds produced by microorganisms, including bacteriocins, cyclic lipopeptides, peptaibols, and defensins (Iralu et al. 2024). These AMP-based molecules enable the inhibition of particular microbial competitors with minimum collateral damage to beneficial members of the microbiome. Bacteriocins are ribosomally synthesized peptides typically exhibiting narrow-spectrum targeting activity against conspecific or closely related strains with high specificity (Sugrue et al. 2024). Due to their proteinaceous nature and nonreplicative mode of action, they represent more stable antimicrobial agents for agricultural applications with reduced ecosystem degradation (Fischer et al. 2024)). Examples include Bac-GM17 from *Bacillus clausii* GM17, ericin S from *B. subtilis* A1/3, and amylocyclicin from *B. amyloliquefaciens* FZB42. Unlike broad-spectrum chemical treatments, most bacteriocins exhibit a narrow host range, enabling precise targeting of plant-pathogenic *Xanthomonas* while preserving surrounding microbiome and environmental integrity (Rooney et al. 2020, Fischer et al. 2024).

Beyond bacteriocins, other AMPs offer narrow-spectrum antifungal activity. Peptaibols, produced by fungi including *Trichoderma* and *Emericellopsis*, have gained research attention for their capacity to specifically inhibit phytopathogens such as *Fusarium oxysporum* and *Botrytis cinerea*.(Shishupala 2025, Shi et al. 2012, Pereira-dias et al. 2023). Diverse AMPs have received immense research interest due to their ability to selectively inhibit specific phytopathogens like *F. oxysporum* and *B. cinerea* (Shi et al. 2012, Pereira-dias et al. 2023). Lipopeptides produced by *Bacillus* and *Pseudomonas* species demonstrate selective activity against specific fungal and bacterial pathogens, while emphasizing reduced toxicity to beneficial microbiome members (Raaijmakers et al. 2010). Although, plant defensins represent another class of narrow-spectrum AMPs with targeted activity against specific fungal pathogens, offering potential for engineering enhanced resistance (Islam et al. 2017). However, AMP application faces critical challenges including production costs, stability issues, potential for resistance development, and off-target toxicity. Further application has been constrained by challenges involving high costs of production, instability, and toxicity to the plant cell (Rooney et al. 2020, Banerji et al. 2022). For example, *Erwinia amylovora*, causing fire blight in apples, demonstrates rapid resistance to phage bioconrol, necessitating constant updating of phage cocktails (Bouazizi et al. 2025). This AMP related efficacy is further hindered by delivery and formulation challenges and complex regulatory approval processes (Wu et al. 2025). In fact, most of these challenges indicate a critical need for more efficient and specific delivery systems as the biocontrol agents.

A novel frontier in targeted microbiome engineering positions BCISs as innovative tools for precise microbial interactions. BCISs represent prokaryotic nanomachines inspired by phage tails, which are competent in loading effector proteins that promote various biological functions by direct delivery into target cells (Nagakubo et al. 2024). The two most intensely investigated mechanisms are the intracellular T6SSs and eCISs (Li et al. 2025b). T6SSs, found only in Gram-negative bacteria, enable direct, contact-dependent target-cell protein delivery through the bacterial cellular envelope. In contrast, eCISs are released into surroundings following cell lysis, with tail fibers binding target cell surfaces, leading to sheath contraction and cell envelope puncturing for effector protein delivery (Zachs et al. 2024). R-type pyocins represent a special type of extracellular contractile agent that primarily acts mechanically rather than as carrier agents, targeting and killing *Pseudomonas aeruginosa* competitors by creating membrane-binding structures that cause proton potential loss, resulting in rapid cell death (Ge et al. 2015). Unlike bacteriophages, eCISs are nonreplicating, DNA free, proteinaceous phage-tail nanostructures that remain stable outside the cell and function independently. Also, potentially evading risks linked to phage replication and evolutionary dynamics in natural environments (Nagakubo 2022). Their mechanical injection bypasses receptor-dependent steps of infection that generally drive rapid phage resistance, while modular architectures allow rapid retargeting by exchanging tail fibers and effector cargos (Kreitz et al. 2023). More importantly, eCISs can be engineered for highly refined host specificity, thus minimizing off-target effects, and their robust protein shells support formulation and delivery in agricultural environments.

## Molecular mechanisms and evolutionary foundations of eCIS targeting specificity

**Figure 2:**
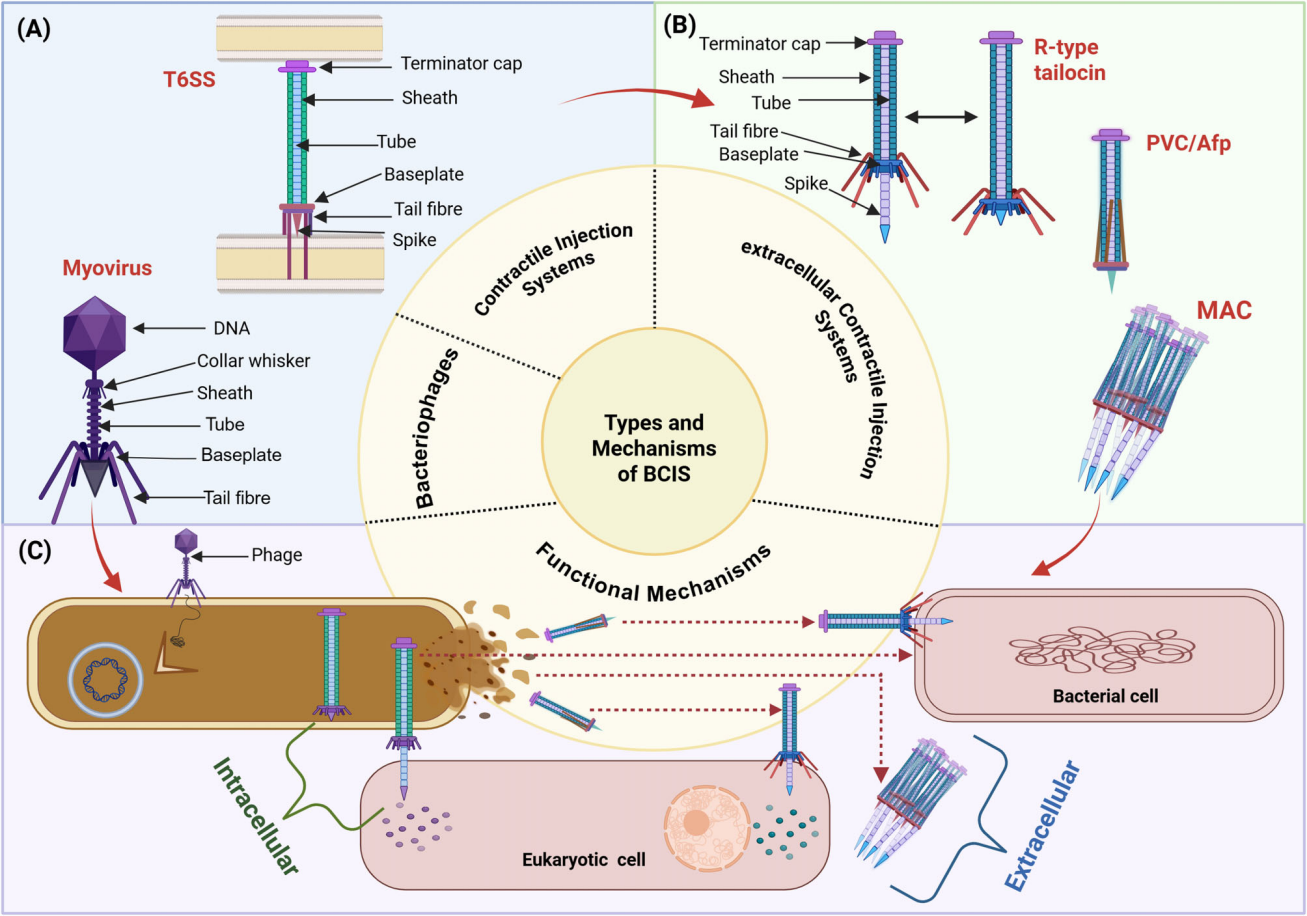
The major classifications, primary targets, and functional evolution of the various contractile injection systems (CISs). (A) CISs are evolutionarily derived from Myoviridae phage tails and are broadly classified into two functional classes: (i) T6SSs, which remain anchored to the bacterial cell envelope for direct effector delivery into neighboring cells, and (ii) extracellular CISs (eCISs), which are released into the environment. (B) extracellular CISs including R-tailocins, PVCs, Afps, and MACs, with contractile tail-like structures (80–180 nm) and MACs form larger arrays (∼310 nm components assembling into 920 nm structures). (C) The functional mechanisms of major eCIS systems can be categorized into three operational modes: (i) phages inject genetic material into bacteria cells (ii) T6SSs deliver effectors through cell contact to prokaryotic/eukaryotic targets, and (iii) upon cell lysis, bacteria release eCIS particles such as R tailocins, which targeting prokaryotic and eukaryotic cells and PVCs, Afps, and MACs which inject effector proteins into eukaryotic host cells.

The targeting specificity of eCISs determined by their unique molecular mechanisms and shaped by their evolutionary origin from bacteriophage tails, which providing precise interactions with selected microbial hosts. Diverse Gram-negative bacteria possess CISs that breach the membranes of neighboring cells to deliver various effector proteins (Wang et al. 2019). These syringe-like macromolecular assemblies are capable of targeting both bacterial and eukaryotic cells. Based on their deployment mechanisms, CISs are primarily categorized into two major categories, including T6SSs and eCIS. The T6SS is a cell-anchored, contact-dependent weapon that injects effectors into adjacent cells (Fig. 2A). In contrast, eCISs are assembled in the bacterial cytoplasm and released into their environment, generally through cell lysis, to act on distant targets (Xu et al. 2022). Also, eCISs have been repurposed by bacteria and archaea as versatile platforms for interspecies communication, antagonism, and cooperation (Peterson et al. 2020). Therefore, this section discusses the three core components that enable this precision including modular physical structure of eCISs, the mechanisms for loading specific molecular payloads, and the genetic determinants that define their host range.

### Structural modularity and contractile dynamics of eCISs

Structurally, eCISs comprise a baseplate, contractile sheath, inner tail tube, distal spike, capping terminator, and specialized tail fibers that mediate target recognition (Geller et al. 2021). Sophisticated eCISs are widely distributed among diverse microbial taxa, and their functional characterization remains limited to four systems, including R-type tailocins, Afps, PVCs, and metamorphosis-associated contractile systems (MACs) (Fig. 2B). All these four eCISs exhibit variability in structural architechture, target recognition, and the cargo-delivery mechanism. The R-type tailocins are DNA-free, phage tail-like nanostructures with a contractile sheath and tail fibers that mechanically disrupt bacterial cells. The PVCs are characterized by their cylindrical body with programmable target cell recognition through tail fibers that used for the transferring protein-based toxins. The Afps are composed of a network of 18 proteins with a contractile sheath and needle, used to inject insecticidal effector molecules into target cells of the insect gut. And MACs are self-assembled into extracellular arrays with tail spikes and baseplates to inject effectors into multiple tubeworm larval targets. This specific structural modularity of eCIS is the key feature that enables its engineerable capability, by understanding these components; we can build custom tools for specific agricultural challenges.

**Figure 3:**
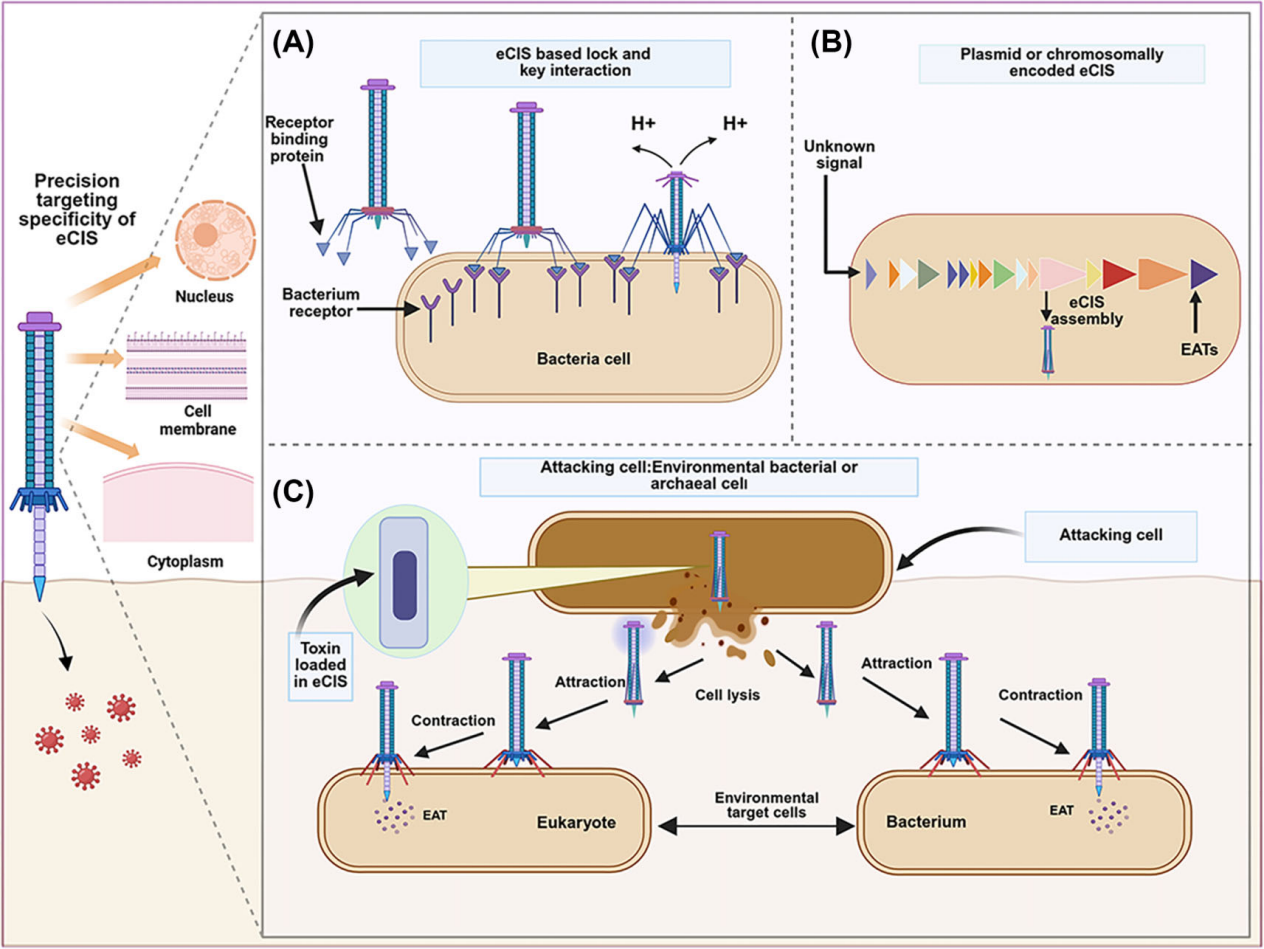
eCIS-based molecular mechanisms governing tail fiber-mediated host specificity and effector delivery. (A) Molecular recognition between eCIS RBPs and specific bacterial surface receptors. This recognition evolves between tailocins RBPs and bacterial surface receptors are governed by lock-and-key interactions at atomic resolution. (B) Intracellular assembly of eCIS machinery (plasmid or chromosomally encoded eCIS) triggered by unknown signals, resulting in effector-loaded eCIS complexes. (C) Preassembled eCIS complexes package cytotoxic effectors (EATs) within their contractile sheath structure. These particles are released through programmed producer cell lysis, a process mediated by holin–endolysin systems that precisely time deployment to maximize target encounter probability. And target cell recognition is mediated by tail fibers, which initiate sheath contraction and effector (EAT) injection, this process typically resulting in target cell death or growth inhibition.

Emerging studies on tailocins, such as R-type pyocins from *P. aeruginosa* represent one of the most well-characterized and earliest discovered eCIS classes. Phage tail-like nanomachines are produced in response to DNA damage, with holin and lysin enzymes triggering cell lysis to facilitate their release (Nakayama et al. 2000, Rajaure et al. 2015). At the molecular level, R-tailocins consist of a sheath encasing a hollow tube that assembles into an elongated helicoidal hexameric structure, terminating in a baseplate bearing multiple tail fibers that mediate target recognition (Ge et al. 2015) (Fig. 2B). In contrast to phages, R-type tailocins are free from a DNA-containing capsid, which provides horizontal gene transfer and helps make them a safer therapeutic option without the risks of unintended genetic recombination (Mei et al. 2025). Also, their simplified structure provides greater stability than phages. They avoid the fragility associated with viral capsids during preparation, storage, and delivery. In essence, target recognition occurs when tailocin tail fibers act as RBPs, binding with high specificity to defined lipopolysaccharide (LPS) epitopes on susceptible Gram-negative cells. And this interaction effectively triggers sheath contraction (Fig. 3A). This contraction drives the inner tube through the target cell envelope, and delivering cytotoxic payloads or causing membrane disruption (Heiman et al. 2023). Tailocins have been identified in various Gram-negative and Gram-positive bacteria, including human, animal, plant pathogens, and environmental saprophytes occupying different ecological niches (Borowicz et al. 2025). Notably, these bacterially encoded tailocins exhibit remarkable modularity, a feature that has been extensively explored in recent engineering studies. For example, by modifying the tail fiber domains of R2-type pyocins, researchers have successfully reengineered their targeting range to include not only various *P. aeruginosa* strains but also pathogenic bacterial species like *Escherichia coli* (Mei et al. 2025). Considering this, the ability to purposefully restructure tailocins indicates their potential as precision antibacterial agents, with promising applications in plant-based microbiome editing and targeted pathogen control. Furthermore, they also overcome important limitations of bacteriophage therapy due to their structural simplicity and lack of a DNA-containing capsid, making them safer and more stable than bacteriophages for field applications.

More broadly, research has highlighted the PVC, as a promising platform for precision protein delivery due to its inherent modularity and programmable targeting capacity. These PVCs are produced by *Photorhabdus* species, which exist as endosymbionts in entomopathogenic nematodes (Sajnaga 2020). While morphologically resemble to phage tails, PVCs feature a reduced wedge region alongside a conserved baseplate and exhibit cylindrical body structure capped distally by a hexamer, that seals and stabilizes the sheath-tube assembly (Jiang et al. 2019). Functionally, these protein complexes penetrate via eukaryotic cell membranes to deliver either native toxins or engineered protein payloads (Fig. 2C). The tail fiber protein Pvc13 plays a important role in determining tropism by mediating interactions with specific cell surface receptors and can be rationally modified to redirect targeting specificity. For example, Kreitz et al. (2023) reprogrammed PVCs to deliver Cas9–sgRNA complexes into human cells by engineering Pvc13 fibers with HER2-targeting nanobodies, achieving greater than above 30% indel frequency proof of programmable genome editing. Moreover, PVCs demonstrate eCIS programmability through modular receptor-binding domains and heterologous payload encapsulation, enabling precision therapeutic delivery and engineered biocontrol applications.

In additionally, the Afp represents a versatile, phage-derived nanomachine with dual roles in insect pathogenesis and programmable biotechnological applications. The Afp gene cluster, located on the pADAP plasmid, includes an upstream lysis cassette encoding endopeptidase, holin, and lysozyme, which are predicted to mediate producer cell lysis and release of Afp particles (Sitter et al. 2024, Hurst et al. 2025; Fig. 2C). It comprises 18 gene products (6.5–263 kDa), with the first 16 open reading frames showing orthology to the PVCs in *Photorhabdus luminescens* TTO1, indicating a conserved structural organization among these contractile injection systems (Vlisidou et al. 2019). Mechanistically, Afp features a contractile protein sheath surrounding an inner tube that is predicted to contain and deliver a toxic payload. For example, Afp delivers the insecticidal effector Afp18 into *Costelytra giveni* larvae, causing rapid feeding cessation consistent with midgut paralysis (Desfosses et al. 2019). Furthermore, the final atomic model of the extended Afp reveals a complex assembly of 11 different proteins: For example, a central tube formed by Afp1, Afp5, and Afp7, an outer sheath polymerized from Afp2, Afp3, and Afp4, a penetration needle composed of Afp8 and Afp10, baseplate incorporating Afp9, Afp11, Afp12, and an apical capping structure formed by Afp16 (Desfosses et al. 2019). This detailed structural and mechanistic characterization provides foundation for engineering targeted biocontrol agents by modifying Afp’s host-recognition and payload-delivery components. Also, *Serratia proteomaculans* produces eCIS particles known as AfpX, and they show faster killing capability than the other Afp-producing strains. Apart from their inherent insecticidal properties, Afp and related phage tail-like nanomachines are promising models for adaptable nano devices with potential applications in biotechnology and medicine, such as targeted delivery systems and immunotherapy. For phytomicrobiome engineering, the ability to modify the host-recognition components of Afp provides a direct route to developing novel bioinsecticides against crop pests that are currently difficult to manage.

Recent findings indicate that, MACs function via coordinated sheath contraction, propelling an inner tube and penetration spike into target cells. MAC particles produced by the marine bacterium *Pseudoalteromonas luteoviolacea*, which represent a highly coordinated eCIS involved in the larval development and metamorphosis of tubeworms (Alker et al. 2020). These MACs provide a conserved mechanism, evolutionarily related to the contractile tails of bacteriophages, whereby sheath contraction drives the inner tube and spike into target cells. Structurally, MACs composed of an inner tube protein that is encased within a contractile sheath and homologous to gp19 from phage T4 and Hcp from the T6SS (Chen et al. 2019). This assembly also includes a tail-spike and a baseplate complex. When bacterial cell lysis, MACs self-assemble into extracellular hemispherical arrays of ~100 units, creating a delivery platform with baseplates facing outward and interconnected by tail fibers. This architecture enables MACs to inject a various types of effector proteins directly into the tubeworm larval tube lumen (Vlisidou et al. 2019). Effectors such as metamorphosis-inducing factor 1 and the nuclease Pne1 have been identified, showing MACs’ dual actions in developmental signaling and defense (Ericson et al. 2019)). Collectively, the precision of eCIS-mediated delivery depends not only modular structural organization and contractile dynamics of MACs but also based on the efficient loading of effectors and their translocation across target membranes. Understanding these effector loading mechanisms represents the next important step in harnessing eCISs as programmable delivery platforms.

### Payload assembly and transmembrane delivery mechanisms

Effector translocation process in eCISs is a finely tuned mechanism that ensures the accurate incorporation of toxic effectors into the particle, thereby enabling their targeted delivery into recipient cells (Fig. 3B). Environmental microbes frequently encode eCIS within operons, typically regulated by adjacent genes such as, RfaH or GerE or BTAD domains, whose activity is modulated through both kinase-dependent activation and response to unidentified signaling molecules (Geller et al. 2021). Structurally similar to the contractile tail of T4 bacteriophages, eCISs are encoded by operons comprising 15–28 genes, with effector proteins typically located at the 3′ end of these operons. Although frequently featuring N-terminal DUF4157 domains or neighboring genes with this domain. The assembled eCIS particles encapsulate toxins within their tubular structure and are released via cell lysis. (Geller et al. 2021) (Fig. 3C). Moreover, studies have demonstrated that effectors possess diverse enzymatic activities in both eukaryotic and prokaryotic cells, commonly resulting in cytotoxic effects. Therefore, PVCs exhibit important adaptations in effector loading that enable them to perform unique roles in particular biological functions, even though they possess conserved structural features with eCISs. For agricultural applications, this diversity in loading mechanisms means that eCISs are not limited to a single type of antimicrobial payload, also opening the platform to delivering a wide range of proteins, enzymes, or toxins to combat different phytopathogens.

Recent progress in PVC serve as a paradigm for eCIS-mediated protein delivery, optimizing a signal peptide (SP)-directed loading mechanism to puncture eukaryotic cell membranes and deliver cytotoxic effectors (Wang et al. 2022). In particular, N-terminal SPs are essential to play a major role in PVC function by directing effector loading into the inner tube as a mechanism validated through Cryo-EM structural analyses and genetic engineering studies (Jiang et al. 2022). By combining genetic engineering approaches, cryo-EM, and translocation assays, SP-guided loading has been demonstrated to enable PVC-mediated delivery of a wide range of prokaryotic and eukaryotic proteins across cell membranes. Although SP-dependent targeting system accommodates macromolecules ranging from bacterial toxins to fungal and plant proteins, emphasizing its exceptional versatility (Jiang et al. 2022). In essential, this PVC can successfully deliver proteins derived from parasites and animal sources, significantly expanding its potential applications. These results collectively suggest that, PVC can be reengineered into a flexible nanosyringe for therapeutic protein delivery, capable of holding variety of cargoes regardless of their size, charge, or place of origin (Vlisidou et al. 2019). Moreover, SP-dependent targeting mechanism thus enables PVC to transport macromolecules from bacteria, fungi, parasites, plants, and animals (Jiang et al. 2022). Engineered PVC systems are now becoming as precision tools for targeted drug delivery, agricultural biotechnology, and synthetic biology applications, due to their exceptional ability to deliver proteins across diverse biological kingdoms. For instance, a PVC system could be engineered to deliver enzymes that degrade fungal cell walls, providing a novel biofungicide strategy, or to deliver proteins that specifically inhibit viral replication in plant-associated bacteria.

In addition to PVC systems, The MACs employ effector-loading mechanism to deliver the metamorphosis-inducing protein (Mif1) into tubeworm larvae via contractile injection (Shikuma 2021). Biochemical studies and cryo-electron tomography have demonstrated that Mif1 is selectively loaded into the MAC tube lumen with the assistance of a chaperone-like protein, JF50_12 605 (Nagakubo 2022). Also, the weak interaction between Mif1 and the tube facilitates its rapid release upon sheath contraction. In this context, electroporation experiments further confirm that Mif1 alone is sufficient to trigger host metamorphosis, demonstrating the precision of effector loading and delivery by the MAC system (Shikuma 2021). While MAC exemplifies the specific transport of a single developmental effector, recent computational advances have revealed extensive diversity among eCIS-associated toxins (EATs).

Utilizing machine learning (ML) approaches, researchers have discovered over 2000 candidate EATs across hundreds of microbial genomes (Steiner-rebrova and Halberg 2025). Discovering new EATs are essential for understanding this specialized microbial secretion system’s varied biological functions. This ML-based classification framework opens new pathways for design of both natural effector proteins and engineered therapeutic payloads and AMPs (Danov et al. 2024). These predictive models also identify key toxicity residues and employ SPs to discover novel effectors. Newly identified EATs like EAT14–EAT17, located downstream of eCIS operons, expand the known toxin repertoire. For example, EAT14 and EAT15 show conserved cytotoxicity in both *E. coli* and *Saccharomyces cerevisiae* species, similar to earlier effectors like EAT1, EAT3, and EAT11 (Danov et al. 2024). Breakthroughs in eCIS engineering for precision protein delivery, when synergized with nature’s divers array of eCIS-optimized EATs, offer a powerful toolkit for promising applications across medicine, biotechnology, and agricultural applications. Moreover, EATs show promising potential for antimicrobial drug development and biocontrol potential against pests, leveraging tail fiber specificity and targeted effector activity for precise applications *(*Danov et al. 2024, Jiang et al. 2022). Collectively, eCISs have evolved as precise molecular mechanisms to ensure targeted effector delivery for both prokaryotic and eukaryotic cells (Fig. 3C). Furthermore, these insights provide a critical foundation for understanding how eCISs achieve selective interactions with host cells, highlighting the importance of dissecting the underlying genetic determinants that govern host range and specificity. These genetic elements, particularly the tail fiber proteins, are the ultimate arbiters of an eCIS’s target and thus represent the primary locus for engineering new functionalities.

### Genetic determinants of host range and specificity

Host range specificity in eCISs is genetically programmed through RBPs, with modular tail fibers that structurally and functionally analogous to phage tail fibers, which determine target cell selection (Heiman et al. 2023). For example, the tail fiber protein Pvc13 in PVCs, play a critical role in host recognition. By replacing its native receptor-binding domain with engineered nanobodies, such as one targeting the human HER2 receptor, researchers have successfully redirected PVCs from insect cells to human cancer cells, demonstrating the modularity and programmability of these systems (Kreitz et al. 2023). Similarly, Afp18 (homologous to Afp13 in *Serratia*) is important for the host targeting and shows sequence homology with adenovirus fibers, suggesting an evolutionary adaptation for eukaryotic cell recognition. Comparative analysis of Afp13 homologs revealed that 76 sequences closely align with viral proteins targeting eukaryotes, while four match with the bacteriophage fibers, indicating that some eCISs retain prokaryotic targets (Geller et al. 2021). Together, eCIS host-range adaptation is facilitated by genetic variation in tail fiber domains, while successful delivery determined by effector compatibility with intracellular environments.

These eCIS systems deliver effector proteins that require precise molecular compatibility with host cellular machinery to induce targeted toxicity. These effectors, located at the ends of eCIS operons, that are released through a unique extracellular contractile mechanism that distinct from other secretion systems (Patz et al. 2019). Effector genes are combined with cognate immunity genes within the same operons, providing self-protection, while maintaining host-specific toxicity an evolutionarily coordinated strategy that shapes host range. Functional analyses have showed that effectors like Pdp1 and Pnf depend on host-specific translocation domains, where single amino acid substitutions can eliminate toxicity without affecting secretion (Wang et al. 2022). Comparative genomics reveal that eCIS-mediated tail fibers frequently acquire genetic modules from bacteriophages, which allows for host range shifts. For example, *Serratia* eCIS tail fibers share approximately over 40% sequence similarity with *Salmonella* phage P22 fibers, indicative of lateral gene transfer (Chen et al. 2019). This modularity extends to effectors, which evolve host-specific features like nuclear localization signals. Furthermore, 13 different toxins have been observed through experimentation to inhibit bacterial or eukaryotic growth, highlighting the versatility of the eCIS machinery.

The evolution of eCIS specificity occurs by genetic mechanisms including, spontaneous mutations, gene loss, and genomic rearrangements within the eCIS loci. Genetic alterations can lead to functional diversification or loss of targeting ability, reflecting an evolutionary arms race between eCIS-producing organisms and their hosts (Heiman et al. 2023). The targeted mutagenesis studies shown, precise genetic control of these traits by showing host specificity can be eliminated or altered by deleting or altering specific genes within the eCIS clusters, especially those encoding structural components or chaperones key for assembly. Also, the presence of accessory genes adjacent to core eCIS genes, such as those encoding hypothetical proteins or virulence regulators, suggests that host specificity may also be modulated by complex genetic networks beyond the primary structural genes (Chen et al. 2019). These findings highlights, genetic determinants of eCIS host range and specificity are multifaceted, involving both core structural genes and accessory factors that coevolve with host targets to optimize molecular recognition and delivery mechanisms. The ecological improvement and evolutionary persistence of eCISs in a variety of microbial and eukaryotic systems are anchored by their genetic modularity and adaptability. By modifying their payloads, structures, and genetic determinants, researchers can overcome the limitations of existing tools and create precision-guided therapies for the phytomicrobiome.

The ecological distribution of eCIS loci reveals a remarkable evolutionary specialization, with significantly enriched in environmental microbes that interact with aquatic hosts, plants, and invertebrates. This pattern of niche-specificity indicates that, eCISs have evolved into specialized toxin-delivery systems that are tailored into particular host–microbe interactions (Geller et al. 2021). Also, these findings position eCISs as evolutionarily refined, genetically modular platforms, in which the combination of adaptable RBPs and specialized effectors enables precise host targeting (Backman et al. 2024b). Their distribution in environmental microbes is a remarkable illustration of ecological adaptation at the molecular level, showing how these systems have adapted for nonmammalian hosts.

## Unlocking the potential of eCIS based precision phytomicrobiome engineering

The phytomicrobiome, known as the second genome of the plant, which plays an essential role in plant health by facilitating nutrient uptake, enhancing stress tolerance, and further conferring effective disease resistance (Singh et al. 2024). Compared to the traditional secretion systems, eCISs are released upon producer cell lysis and can proceed on distant microbial targets, making them as suitable for spatially controlled interventions (Xu et al. 2022). In specifically, their programmable nature facilitates selective targeting of phytopathogens, while sparing beneficial microbes, offering an ecofriendly substitute for broad-spectrum chemical pesticides (Becker et al. 2022). Therefore, the following section focuses on highlighting the potential of reprogrammable eCISs as next-generation tools for sustainable plant pathogen suppression and microbial community engineering.

### Targeted biocontrol of agricultural pests via eCIS toxin delivery

Recent studies illustrate that, nearly 40% of the world’s crops are destroyed due to various pests and disease problems each year, highlighting the crucial need for more effective pest control solutions (Wyckhuys et al. 2025). Recent research has provided insights into the advancement of eCISs in biological pest control, offering specificity against agricultural threats (Steiner-rebrova and Halberg 2025). These sophisticated molecular machines function with remarkable precision by delivering targeted effector proteins directly into pest cells. For example, the Afp produced by *Serratia entomophila* shows precision through selectively infecting grass grub larvae and causing amber disease. Also, this disease condition causes cessation of feeding and leads to systemic collapse of the relevant pests (Ortiz and Sansinenea 2023). The PVCs use toxins that specifically impair insect immune systems, and this system works by injecting toxins that disrupt insect cellular functions, leading to rapid death or behavioral changes (Belyy et al. 2023). Unlike conventional pesticide applications, which often disrupt entire ecosystems, eCISs are capable of distinguishing between pests and beneficial organisms, thus protecting bees and soil microbiota (Daisley et al. 2022). Target specificity is provided by tail fibers that can recognize unique surface markers on target cells, ensuring selective elimination of particular insect pests (Ibarguren et al. 2025). Therefore, these precision targeting strategies significantly enhances environmental safety but also reduces the susceptibility to pest resistance, addressing a critical concern in modern agriculture.

**Figure 4:**
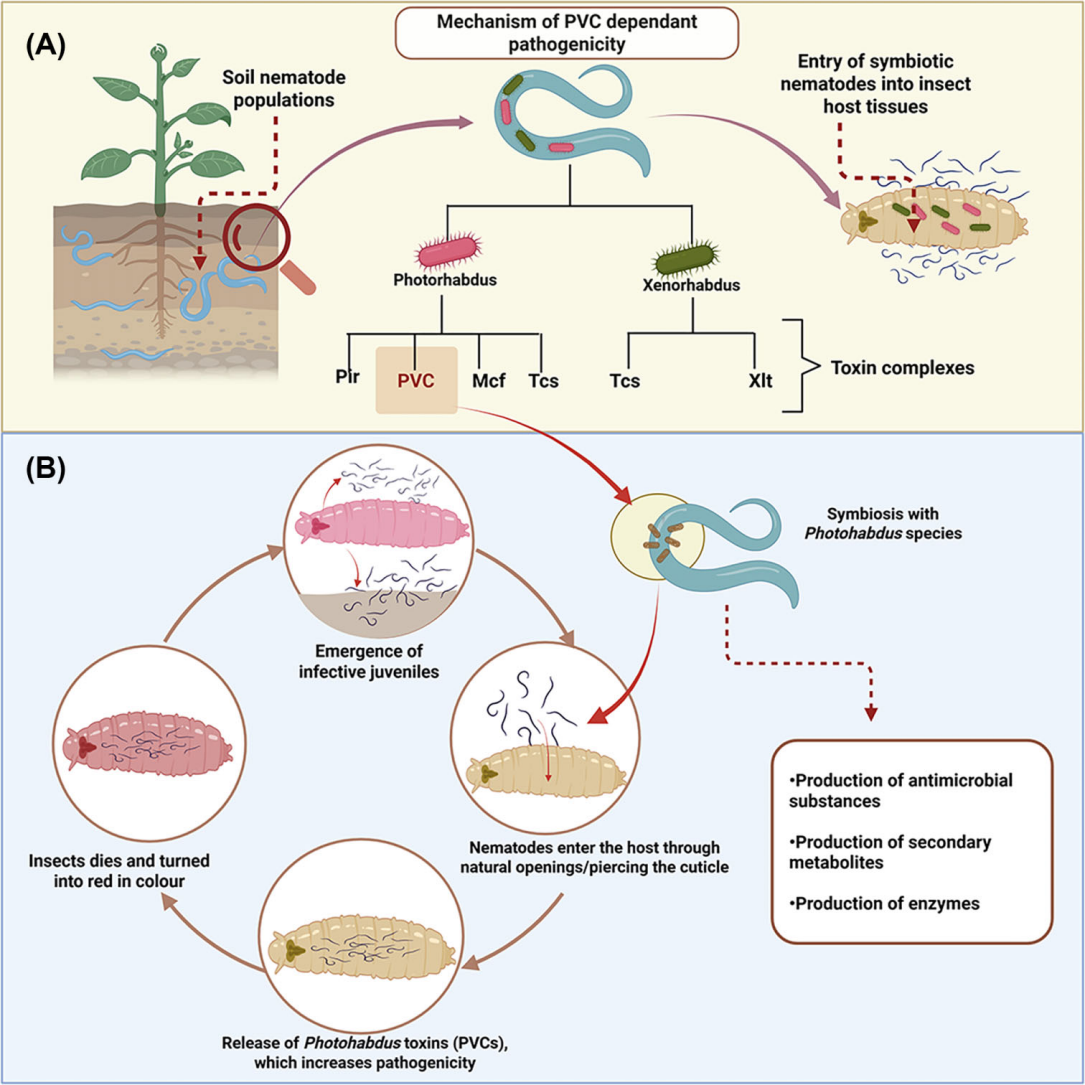
Mechanism of PVC-dependent insect pathogenicity in *Photorhabdus* and nematode symbiosis. (A) Major toxins complexes for virulence determinants, including PVCs and other toxin complexes (Pir, Mcf, and Tcs), produced by entomopathogenic bacteria. (B) The crucial steps in the infection process are invasion of insect hosts by nematodes, releasing symbiotic *Photorhabdus*, which excretes PVC toxins, disrupting host immunity, thus leading to rapid insect death. Essentially, various antimicrobials and metabolites ensure host colonization and nematode reproduction as an emerging tool for targeted biocontrol of pest insects.

In addition, *Photorhabdus* bacterial symbionts of entomopathogenic nematodes that employ different mechanisms of toxin delivery in insect target hosts like coleopteran insect pests and lepidopteran insect larvae (Abd-elgawad 2021) (Fig. 4B). This PVC has the potential for delivering proteins into insect cells, hence contributing to the broad insect target pest control that makes them potential bioinsecticides. Although PVCs employ a contractile nanosyringe for direct mechanical injection, avoiding cellular uptake barriers to deliver effectors with high precision, unlike they different from common bacterial toxins that rely on diffusion or endocytosis (Lu and Cai 2023). This targeted delivery system shows great potential for use in agriculture, providing a targeted alternative to broad-spectrum chemical pesticides in controlling insect pests.

The breakthrough studies centered on the application of bioinformatics approaches aimed at the discovery of new variants of eCIS and the design of their effector proteins for diverse applications (Danov et al. 2024). Despite the continued limitations of mass production, based on the self-destructive nature of bacterial hosts and advances in synthetic biology and fermentation technologies are allowing the development of scalable solutions. Since eCIS progresses from laboratory studies to field implementation, this has a great potential to redefine sustainable agriculture by combining ecological preservation with effective pest management strategies (Strathdee et al. 2023). Overall, eCISs consist of transformative approach in crop protection, balancing highly effective pest management with ecological integrity. Based on this potential, recent efforts have begun exploring the reprogramming of eCISs as precise tools for modulating plant-associated microbial communities.

### Broader microbiome modulation by shaping microbial communities

As recent findings indicate, eCISs represent a versatile class of phage tail-like nanomachines that use to deliver diverse effector proteins with remarkable precision and targeted specificity. Expanding on their distinct method of deployment, these eCISs can act as “free-floating” syringes that locate and inject distant targeting cells in a complex environment such as the rhizosphere. This ability is essential for the application in modulating plant microbiomes (Gallegos-Monterrosa and Coulthurst 2017, Lin 2024). Also, reprogramming of eCISs in light of key advances emphasizes their conserved engineering of tail fibers to reach host specificity and customization of the payloads to suit specific biotechnological applications. In this regard, the tail fibers are extremely diverse, having fast-evolving glycan- and protein-binding domains that specifically recognize microbial surface molecules, such as LPSs, exopolysaccharides, and various types of glycan motifs. Therefore, eCISs are able to selectively target pathogenic microbes from beneficial microbiota within complex plant-associated microbial communities. Moreover, advances in Cryo-EM approaches have positioned it as a transformative platform for high resolution insight into structures of eCISs, their entire assembly, and their targeting and effector delivery systems.

**Figure 5:**
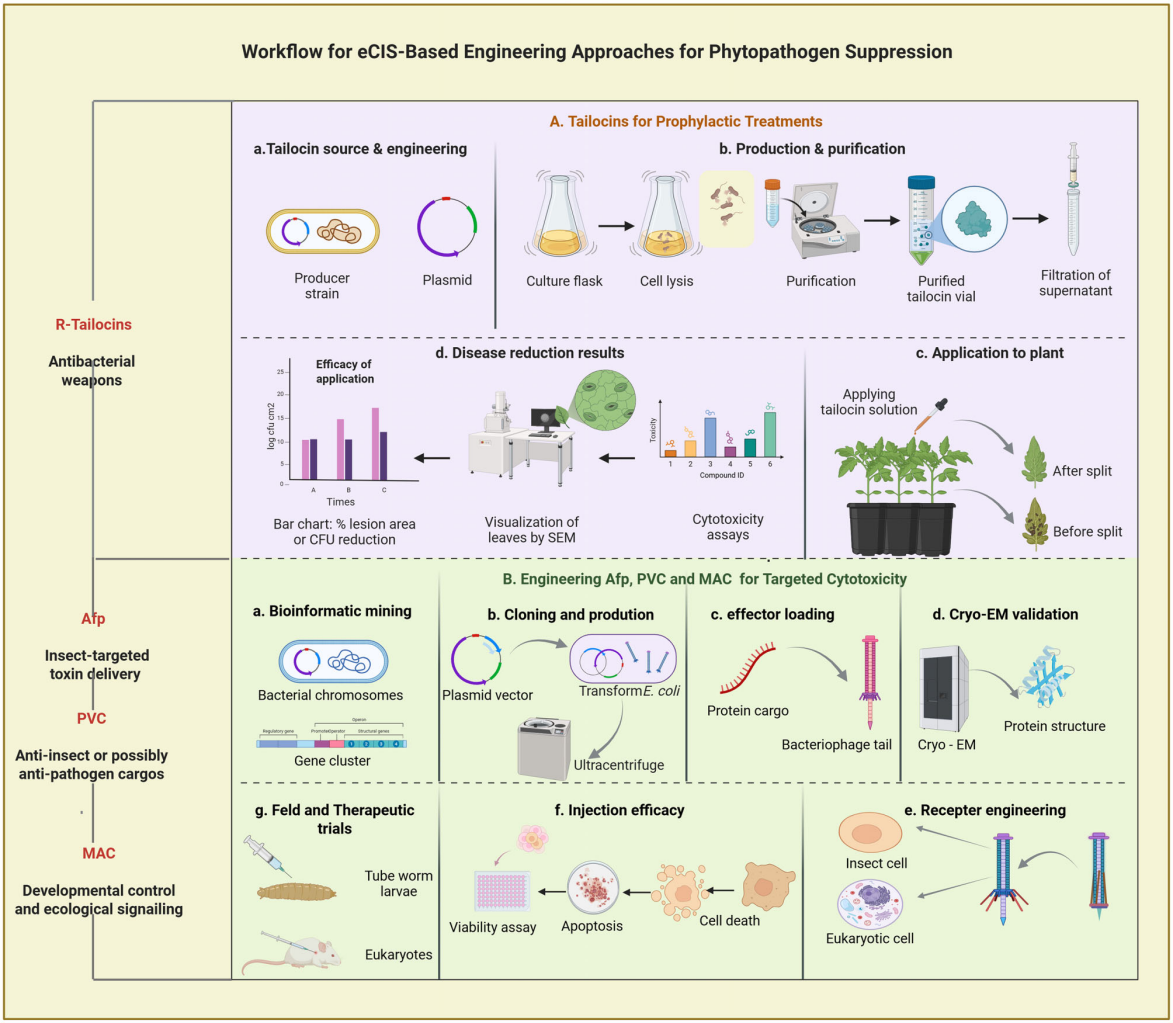
Comprehensive workflow for eCIS-based engineering approaches for phytopathogen suppression. (A) Tailocins for phytopathogen control, schematic overview illustrating the use of R-type tailocins as antibacterial agents in plant disease management. (B) Engineering Afp, PVC, and MAC systems for targeted cytotoxic delivery, conceptual framework describing the engineering of contractile injection systems for insect-targeted or antipathogen applications.

The Cryo-EM and genomic studies have revealed that eCISs have an evolutionarily conserved structure consisting of phage tails, comprising sheath, nanotube, and baseplate components. This evolutionarily conserved structural platform provides modular adaptations, particularly within RBPs and their associated chaperones that are specific to these modules. This ensures that even as functional modules like RbPs can be swapped or modified to alter host specificity (Fig. 5B). For example, in *Pseudomonas syringae* tailocins, engineered RbPs have enabled interspecies killing of pathogens such as *Xanthomonas* and *Salmonella* through specific interactions with tailored LPSs (Weaver et al. 2022). Further, DNA inversion and modular exchange methods have altered host specificity in R-tailocins from *Xenorhabdus bovienii* and *Pectobacterium carotovorum* (Heiman et al. 2023). The activation mechanism of R-type pyocins is precisely triggered by ligand recognition via tail fibers, resulting in efficient, single-shot killing of targeted bacteria (Dunne et al. 2021). All these features demonstrate the capability of using engineered eCISs as potent and specific antimicrobial agents. Because of their adaptability, RbPs serve as the essential tool for precisely modifying the plant microbiome, which allowing targeted reduction of pathogens while maintaining beneficial microbial communities.

Recent advancements demonstrate that adaptability of eCISs extends to their payloads, which can be precisely engineered for the targeted delivery of therapeutic or antimicrobial proteins where they are needed (Fig. 5B). This enables efficient loading and injection of range of toxin cargos into target cells by combining modified effector domains and changing N-terminal signaling peptides (Jiang et al. 2022). For this purpose, thousands of EAT genes from various bacterial phyla have been identified through bioinformatic surveys and many of which demonstrate proven antibacterial or antifungal properties (Chen et al. 2019). A prominent example includes the EAT10, a peptidoglycan hydrolase, exhibits heightened toxicity within the periplasm of *E. coli*, suggesting its potential utility against Gram-negative pathogens by compromising cell wall integrity. Similar to this, the ld-transpeptidase EAT6 specifically suppressed the growth of *E. coli*, most likely by interfering with the peptidoglycan cross-linking. The RES-like EAT5 toxin, an NAD+ phosphorylase, is neutralized by its cognate immunity protein, highlighting a toxin–antitoxin system that could be engineered for precise bacterial growth inhibition (Geller et al. 2021). EAT12, a putative toxin that resembles the nucleoporin, provides prokaryotic-specific, sparing eukaryotic cells and further enhances its potential as a narrow-spectrum antimicrobial agent. The frequent association of DUF4157 with EATs like EAT4 and EAT7 further suggests a role in toxin loading or activation, presenting a novel target to disrupt eCIS-mediated bacterial competition (Geller et al. 2021). Together, these diverse and precisely engineerable effectors show the immense potential of eCISs as customizable platforms for developing targeted, efficient, and sustainable antimicrobial strategies in both agricultural and clinical contexts.

Distinct from other eCISs, the PVC systems determine host tropism, and its engineering enables predictable retargeting to novel hosts. This capability has been demonstrated by successful tail fiber exchanges in Afp particles, which altered fiber length and host specificity without compromising particle integrity (Bhardwaj et al. 2021). Further, these advances emphasize reprogrammable eCISs as efficient nanomachines for sustainable engineering of plant-associated microbial consortia.

### A toolkit of natural and engineered eCISs for plant health

These eCISs deliver toxic effector proteins directly into pathogenic microbes with high specificity, hence reducing off-target effects (Backman et al. 2024a). Especially, extracellular release of the eCIS particles can take a lead role in acting at a distance, thus evading limitations associated with contact-dependent secretion systems, such as T6SS and T4SS (Souza et al. 2015, Lin 2024). Such engineered CISs able to suppress plant diseases by delivering lethal effectors directly into target pathogens through programmable RBPs as demonstrated in *P. syringae* tailocins (Baltrus et al. 2022). Through their selective killing of pathogens to preserve beneficial microorganisms, eCISs improve plant productivity by promoting health and reducing competition for soil nutrients. These attributes show eCISs as promising biocontrol platforms that align with the goals of sustainable agriculture by offering precise, effective, and environmentally friendly alternatives to chemical pesticides.

#### R-Type tailocins in phytomicrobe defense

For successful survival within highly competitive microbial environments, bacteria employ very efficient mechanisms such as resource sequestration and the production of toxins (Vacheron et al. 2021, Iqbal et al. 2023). The most specific mechanisms among these include tailocins, which can kill specific and closely related bacterial strains (Backman et al. 2024a). Although a single producer cell can release dozens to hundreds of tailocins, enabling it to eliminate a large population of competing cells with remarkable efficiency (Backman et al. 2024a). Therefore, the combination of high killing efficacy, environmental durability, and precise strain specificity positions tailocins as exceptional agents for developing targeted antimicrobials in agriculture. Recent studies have provided insights into the R-tailocins exclusively target bacterial cells by destabilizing their membranes, functioning without the injection of effector molecules (Carim et al. 2021a) (Fig. 5A). Live-cell imaging reveals that R-tailocins are assembled centrally and released by explosive lysis, dispersing them over tens of micrometers. For example, tailocins, a diverse group of eCISs, represent tail-derived bacteriocins exemplified by the well-characterized R-type pyocins of *P. aeruginosa* (Vacheron et al. 2021). Live-cell imaging reveals that R-tailocins are assembled at the cell center, trafficked to the poles, and released through explosive cell lysis, a mechanism that facilitates their dispersal over tens of micrometers to target cells (Vacheron et al. 2021). Furthermore, following SOS-mediated induction and cell lysis, released R-tailocins bind susceptible bacteria via tail fiber interaction with specific surface receptors, including LPSs. Initiation of contact triggers irreversible contraction, propelling the core through the cell wall in order to depolarize the target cell membrane, resulting in killing of the target cell (Baltrus et al. 2025). The initial R-tailocin-type eCISs were discovered based on induction, purification, and efficacy assessment on a very narrow range of bacterial strains (Heiman et al. 2023). However, more recent studies have expanded this scope, revealing complex phylogenetic and ecological interactions among susceptible taxa.

Compelling evidence from Principe et al. (2018) has shown that tailocins from the *P. fluorescens* SF4 strain, applied through foliar sprays, can reduce the severity and incidence of bacterial spot in tomato caused by *Xanthomonas vesicatoria*. In addition, SF4c is a nontoxic and stable alternative for copper-based pesticides because it retains functionality under various stresses and is not toxic to human cells (Príncipe et al. 2018). An added advantage of tailocin-based targeting is that resistance mutations often incur significant fitness costs, frequently leading to reduced virulence in plant pathogens. Other experiments focused on the prophylactic treatment with R-type tailocins functioning effectively to prevent foliar infections caused by (Backman et al. 2024b). Other recent studies focus into prophylactic treatment using R-type tailocins, which effectively inhibited foliar infection caused by *P. syringae* pv*. syringae* B728a in *Nicotiana benthamiana* plants (Baltrus et al. 2022). These results of the prophylactic test of tailocin are representative of a highly effective and selective method of plant protection for the prevention of bacterial infection. Though prior studies have firmly established the intraspecific killing spectrum of these tailocins against closely related strains, the tailocins produced by *P. syringae* USA011R also have significant interspecific killing capability. For instance, tailocins from this strain exhibit interspecific activity against the phytopathogens *E. amylovora* and *Xanthomonas perforans*, and even a specific *Salmonella* species (Baltrus et al. 2022). Therefore, this cross-species killing activity defining the molecular basis of host range is the key to engineering tailocins with precisely tailored killing spectra.

The global shift to sustainable agriculture fuels the adoption of biopesticides, which ensure less environmental effect and high standards in food safety. This becomes important when controlling seed-borne diseases in rice, which is highly dependent on bacterial pathogens such as *Burkholderia glumae* and *Burkholderia plantarii*. One particular example has been BglaTNB6 synthesized by *Burkholderia gladioli* NB6, which has emerged as a potential biocontrol agent. Recently, it has gained attention as a biocontrol agent due to its selective inhibitory activity against pathogens such as *B. glumae* and *B. plantarii* (Kouzai et al. 2024). Ecological function is also usually rarely studied for their natural habitat of plant pathogenic bacteria occurring under agricultural ecosystems with dynamic environmental stresses. Thus, the SRP, which includes *Pectobacterium, Dickeya*, and *Musicola* species, provides a model for tailocins ecology studies across water, soil, plants, insects, and agricultural milieus (Borowicz et al. 2025). One of the most characteristic examples is the R tailocin of the pathogen *Dickeya dadantii* 3937—dickeyocin P2D1—which displays a highly stable environment by selectively killing the *Dickeya* genus of pathogens while sparing *Pectobacterium* spp. and *Caenorhabditis elegans*. In addition, *Pseudomonas chlororaphis* employs a unique, multifaceted R-tailocin system which featuring two evolutionarily distinct particles and a tail fiber “switching” mechanism to enhance its killing spectrum (Dorosky et al. 2018). This system enhances rhizosphere competitiveness loss of tailocins reduced *P. chlororaphis* persistence against native microbiome, showing niche-specific adaptation. Although these systems are prime examples of eCIS-based structural accuracy, related effector toxins further increase their functional diversity.

Recent studies have unraveled that tailocins, repurposed phage-derived nanomachines, are conserved in *Pseudomonas viridiflava* metapopulations and play an important role in strain-specific competition within the plant environment. This long-term maintenance of distinct tail fiber-LPS variants points to strong selective pressure and evolutionary stability, offering insights into the mechanisms underlying microbial specificity. For example, this natural tailocin diversity offers a template for the rational design of targeted “tailocin cocktails” with goals for the modulation of the phytomicrobiome for sustainable control of pathogen populations. These insights collectively demonstrate how tailocins balance narrow-spectrum targeting and adaptable killing ranges.

#### Engineered eCIS platforms for plant health enhancement

Most importantly, both the Afp and PVCs are eCISs that report significant indirect contributions to modulate the plant microbiome as well as microbial and insect populations impacting plant health (Fig. 5B). These phage tail-like nanomachines are produced by bacteria living in soil and plant-associated environments, where they influence microbial community dynamics and plant–microbe interactions. A precise example of this is the PVCs which encoded by *Photorhabdus* species, are eCISs that deliver diverse protein toxins into insect immune cells, causing immune suppression and insect mortality (Yang et al. 2006). Although, these bacteria are symbionts of entomopathogenic nematodes that infect soil insects. The death of insect hosts releases nutrients and microbial biomass into the soil, which promotes microbial replacement and alters the composition of the rhizosphere microbiome. Moreover, these PVCs can be engineered to deliver various protein effectors, suggesting potential applications in targeted manipulation of plant-associated microbes or pests. By controlling insect populations and influencing soil nutrient dynamics, PVCs indirectly modulate the plant microbiome and promote plant health.

Amber disease, a highly host-specific condition affecting only the indigenous New Zealand grass grub (*C. giveni*), is caused by various strains of the bacteria *S. entomophila* and *Serratia proteamaculans*. Specifically, Afp, produced by *S. entomophila*, targets insect pests such as the New Zealand grass grub by injecting toxins that cause feeding cessation and ultimately result in death (Hurst et al. 2007). These Afp particles, indirectly benefit plants by reducing crop damage and shifting the ecological niche of the soil microbiome by suppressing the numbers of insect herbivores. Thus, insect herbivory reduction might shift the rhizosphere microbial community structure due to changing root exudation and nutrient cycling patterns. Conversely, applying genetic and bioinformatic approaches, scientists identified a novel kind of tailocin-a toxin-delivery system, called AfpX-in the bacterium *S. proteamaculans* AGR96X. The pathogen *S. proteamaculans* AGR96X kills grass grub and manuka beetle larvae in 5–12 days via hemocoel invasion, drastically faster than the months-long timeline of *S. entomophila*. This unique Afp tailocin variant acts by delivering effector proteins to the grass grub gut, inducing immediate feeding cessation. Furthermore, structurally defined nature of two tail-length termination proteins, the AfpX tailocin displays dual larval toxicity, positioning it as a potent *S. entomophila* alternative for pasture pest control. This Afp system thus acts as a biological control agent, shaping the plant-associated microbiome by controlling insect vectors that influence microbial dispersal and plant health.

The eCISs are primarily found in microbes associated with a wide range of terrestrial and aquatic hosts, including plants, insects, annelids, protists, fungi, fish, sponges, and molluscs (Geller et al. 2021). Specifically, eCISs have the potential in natural applications including *Xenorhabdus* and *Photorhabdus* species, which are enteric symbionts of *Steinernema* and *Heterorhabditis* nematodes (Fig. 4A). When such kind of bacteria enter insect hosts, they can produce multiple antibacterial, antifungal, and insecticidal compounds (Sajnaga 2020). For example, *Xenorhabdus szentirmaii* was found to be more effective against fungal pathogens like *Cryphonectria parasitica* than *Photorhabdus* species, showing considerable variation in interspecies antimicrobial activities (Cimen et al. 2021). Altogether, their specific targeting of certain pathogens, programmability, ecological compatibility, and proven efficiency against a wide range of plant systems makes them promising prospects for sustainable biocontrol.

In addition, eCISs encode a diverse array of effector protein types with specialized antimicrobial functions. These EATs widely known as a crucial functional layer of eCIS-mediated interactions and provide strong antimicrobial activity through highly specialized mechanisms of action (Lin 2024). Among these effectors, EAT10 shows the capacity of eCISs to eliminate competing bacteria through cell wall degradation process. These toxins are accompanied by cognate immunity genes that protect the producer strain from self-intoxication. Recent studies have highlighted that at least 13 EATs capable of killing model organisms such as *E. coli* and *S. cerevisiae*, with certain toxins exhibiting enhanced toxicity in the periplasm (Geller et al. 2021). This finely tuned delivery and activation mechanisms have been optimized for intra or interspecies antagonism. Notably, specific effectors and their precise delivery systems show promising potential in harnessing eCISs as targeted biocontrol agents.

## Challenges and limitations

Recent crop-protection approaches remain suboptimal, being heavily reliant on chemical antimicrobials such as copper compounds and conventional antibiotics such as the streptomycin. Their widespread application has been linked to significant environmental and ecological impacts (Verhaegen et al. 2023, Yu et al. 2023). In contrast, eCIS-derived structures offer an environmentally more compatible alternative to pesticide application, as their precise targeting significantly diminishes the possibility of off-target effects on plant or soil microbiomes. Therefore, eCIS-based biocontrols have the potential to manage phytopathogens precisely; however there are still important issues that need to be resolved before they can be widely used in agricultural adaptations.

As a first step, environmental monitoring protocols are required due to ecological impact and safety concerns, which require for a thorough evaluation of particle persistence in soil ecosystems and potential off-target effects on beneficial microbiota and nontarget organisms. For example, R-type tailocins enhance the survival and persistence of *P. chlororaphis* in the wheat rhizosphere but are differentially effective depending on the composition and activity of the native soil microbial community (Dorosky et al. 2018). These findings highlight the need for careful environmental monitoring to determine how long R-type tailocin particles may persist in soils and to determine potential unintended effects on other beneficial microbes. Rapidly evolving tailocins can cause once-potent variants to lose killing activity within a few generations, a variability that creates a limitation by reducing confidence in their long-term sustainability and reliability (Backman et al. 2024b).

The second major challenge is suicidal lysis mechanism in the context of eCISs. This generates major difficulties in scaling up the process of mass production and developing innovative techniques of fermentation for effective and efficient solution. Specifically, tailocin release occurs exclusively through suicidal cell lysis, eliminating the producer despite inherent resistance, thereby rendering tailocin deployment an energetically costly and competitive strategy reliant on the programmed sacrifice of a subset of cells (Carim et al. 2021). In addition, the production and release of eCISs should be precisely regulated to ensure their target activity and to avoid cell death at a population-wide scale for the purpose of suicidal eCIS deployment. Only a small fraction of bacteria naturally produce and release R-tailocins, indicating that eCIS deployment must be tightly regulated to prevent excessive population-wide fitness costs.

Third, there is a challenge related to pathogen evolution, as surface receptor modifications or effector detoxification threaten long-term efficacy. This problem is especially significant because the rapid evolution of resistance often through simple modifications to LPS, results in a short adaptive “half-life” for a tailocin variant, causing its efficacy to decline quickly (Carim et al. 2021). However, the emerging potential of resistance to the tailocin via the modification of the LPS is associated with a pleiotropic cost, which affects the bacterium generally and even leads to hypersensitivity to antibiotics. Furthermore, the persistence of diverse tailocins in nature indicates that their effectiveness is highly context-dependent, fluctuating with environmental, host, and nutrient conditions. Proactive solutions like preparation for multiplexed eCIS cocktails targeting several receptors, are essential for the durable defense (Backman et al. 2024b). As a fourth step, current eCISs have proven effective against bacterial targets, their ability to target fungi and oomycetes remains hampered by the continued poor understanding of both cell wall penetration and tail fiber–receptor interactions. This combination of challenges indicates both the highly prospective character of eCISs and the necessity for multidisciplinary research aimed at the successful use of eCISs.

## Concluding remarks and future perspectives

Previous studies strongly suggested that CISs have emerged over the past decade as a revolutionary approach in microbiome engineering, particularly eCISs, represents a paradigm shift in phytomicrobiome engineering. Beyond the broad-spectrum activities of chemical pesticides and the stability problem related with bacteriophages, the eCISs offer an unprecedented combination of high target specificity, robust environmental stability, and inherent modularity. For example, tailocins show great physical stability, including a few R-type samples can display biocidal action for at least 24 h at various temperature ranges of ~4°C–42°C, while they can remain effective for many months at 4°C–8°C in simple buffers (Sobolewska et al. 2025). Similarly, eCISs can preserve their stability and efficacy for at least 24 h at either environmentally or buffered specifications, while they can last for many weeks to months at refrigerated to frozen temperatures (Geller et al. 2021). As discussed in this review, the structural approach derived from their bacteriophages enables the exact delivery of tailored molecules for the elimination of pathogens without affecting beneficial microbe communities. Hence, eCISs have the capability of using innovative approaches for the shaping of phytomicrobial communities that increase crop productivity. The genetic plasticity of their receptor-binding domains provides a direct pathway for engineering novel targeting capabilities.

Amongst CISs, eCISs have garnered special attention because of their distinct modular architecture, effector-loading strategy, and host-targeting potential. These programmable nanomachines are released extracellularly via suicidal lysis of the producer cell, enabling them to act at a distance and overcome several limitations imposed by contact-dependent secretion systems such as the T6SS (Böck et al. 2017). Furthermore, the designed tail fibers of eCISs make it feasible for detect and bind particular surface molecules of microbes, such as LPSs and exopolysaccharides. This was demonstrated in plant pathogens, as the eCISs are known to have the ability to target specific locations with toxic compounds without harming beneficial symbionts. Their characteristic of swapping or engineering the tail fibers of the eCISs will enhance flexibility toward host ranges, thus making eCISs the next generation of biopesticides that are highly effective, precise, and ecologically safe.

For looking ahead, the next decade is expected to see the rapid progresses in eCIS technology driven by the fusion of synthetic biology with computational modeling and high-throughput screening technologies. Future research ought to involve overcoming issues and challenges pointed out above. Key efforts will focus on the developing probiotic bacterial chassis, like *B. subtilis*, as *in situ* “bioreactors” for the localized production and delivery of eCISs in the rhizosphere, thereby bypassing downstream manufacturing and stability constraints. This approach is encouraged by evidence showing that eCIS and R-type tailocin production can be reliably induced through environmental or chemically mediated DNA-damage signals including UV irradiation, oxidative stress, or SOS-activating agents, which may be manipulated or engineered for controlled activation directly within the plant-associated environments (Heiman et al. 2023). Nonetheless, ML and AI-assisted protein design will continue to accelerate the discovery of novel effector toxins and chimeric tail fiber design to refer resistance and target range. Successful deployment of eCISs will depend on integrating these nanomachines into integrated pest management programs. By integrating crop protection with ecosystem health, eCIS-based technologies have the potential to become a cornerstone of sustainable agriculture, enabling a future where we can precisely edit the phytomicrobiome for enhanced resilience and productivity.
